# Ecology and Geography of Plague Transmission Areas in Northeastern Brazil

**DOI:** 10.1371/journal.pntd.0000925

**Published:** 2011-01-04

**Authors:** John Giles, A. Townsend Peterson, Alzira Almeida

**Affiliations:** 1 Biodiversity Institute, University of Kansas, Lawrence, Kansas, United States of America; 2 Departamento de Microbiologia, Centro de Pesquisas Aggeu Magalhães, Cidade Universitaria, Recife, Brazil; Centre Suisse de Recherches Scientifiques, Côte d'Ivoire

## Abstract

Plague in Brazil is poorly known and now rarely seen, so studies of its ecology are difficult. We used ecological niche models of historical (1966-present) records of human plague cases across northeastern Brazil to assess hypotheses regarding environmental correlates of plague occurrences across the region. Results indicate that the apparently focal distribution of plague in northeastern Brazil is indeed discontinuous, and that the causes of the discontinuity are not necessarily only related to elevation—rather, a diversity of environmental dimensions correlate to presence of plague foci in the region. Perhaps most interesting is that suitable areas for plague show marked seasonal variation in photosynthetic mass, with peaks in April and May, suggesting links to particular land cover types. Next steps in this line of research will require more detailed and specific examination of reservoir ecology and natural history.

## Author Summary

We analyzed the spatial and environmental distributions of human plague cases across northeastern Brazil from 1966-present, where the disease is now only rarely transmitted to humans, but persists as a zoonosis of native rodent populations. We elucidated environmental correlates of plague occurrences by way of ecological niche modeling techniques utilizing advanced satellite imagery and geospatial datasets to better understand the ecology and geography of the transmission of plague. Our analysis indicates that plague foci in Brazil are indeed insular as previously suggested. Furthermore, distribution of such foci are likely not directly dependent on elevation, and rather are contigent on climate and vegetation. Seasonality of zoonotic plague transmission is linked to variations of these ecological parameters- particularly the increase in precipitation and primary production of the rainy season. Spatial analysis of transmission events afford a broad view of potential plague foci distributions across northeastern Brazil and indicate that the epidemiology of plague is driven by a dynamic array of environmental factors.

## Introduction

Plague arrived in Brazil during the Third Pandemic, in October 1899, imported by ship traffic to Santos, in São Paulo state, and was rapidly diffused to other coastal cities. By 1906, it had dispersed by means of land and sea commerce more broadly, and had become established in native rodent populations, particularly in the northeastern sector of the country [1], [2]. Nonetheless, records of plague in Brazil are sparse through the 1920s, making detailed tracking of the pattern of spread of the disease in the region difficult or impossible [1], [3].

Only by around 1936 were data on plague and its control in Brazil regularly collated and archived. Based on analyses of these data (for 1936–1966), Baltazard [1] identified numerous distinct plague foci occurring in different environmental contexts, a viewpoint that was updated by Vieira & Coelho [4], based principally on elevation. These foci appear to exist independently of one another in time and space [1], and overall numbers of human cases varied from 20 to 100 until the 1970s. Since that time, all of the foci entered a period of relative inactivity, with few or no human cases [5], [6], [7], [8]. The last significant outbreak in Brazil was in the late 1980s in Paraíba [9].

The purpose of this contribution is to present a first range-wide analysis of the geography and ecology of plague transmission in northeastern Brazil using tools drawn from the emerging field of ecological niche modeling, which is beginning to see application to plague biology [10], [11]. Although no recent plague transmission to humans has been recorded in this region, plague remains as a zoonosis across much of northeastern Brazil [12], making a thorough understanding of its geographic distribution an ongoing priority. Here, we marshal new tools from quantitative biogeography in the form of ecological niche modeling approaches, which related known points of occurrence to raster geospatial GIS data layers to estimate the ecological niche of a species or other biological phenomenon, such as transmission of a disease [11]. The result is both a spatial prediction of areas of potential transmission and a first-order evaluation of environmental correlates of plague transmission in northeastern Brazil.

## Methods

### Input Data

#### Plague case occurrences

We drew occurrence data from the Serviço Nacional de Referência em Peste do Centro de Pesquisas Aggeu Magalhães, Recife, Pernambuco. The data cover the period 1966-present, and represent only cases with laboratory confirmation by means of bacteriological examination or serological testing [6], [7], [8], [9], [13].

Case-occurrence data were referenced spatially to the particular town or ranch where the affected person lived, which we georeferenced by means of referring to municipal maps at diverse scales published by the Instituto Brasileiro de Geografia e Estatística. Of the total of 203 laboratory-confirmed plague cases, which fell into 157 distinct localities, 120 localities could be georeferenced with a spatial precision of 3 km or finer, and most to within 500–1000 m.

#### Environmental data

We sought fine-resolution geospatial data characterizing environmental variation across the landscapes of northeastern Brazil for the time period of interest. We estimated potential risk areas for plague case occurrences based on indirect, landscape-scale measures that are likely correlates of the actual environmental factors associated with the disease-vector-host interactions that affect the ecology of this disease[14]. In particular, we focused on correlates of key environmental dimensions related to precipitation, temperature, vegetation, topography and land use, so we explored two principal environmental data sets. First, we chose imagery from the Advanced Very High Resolution Radiomater [AVHRR; 15] satellite, in particular using multitemporal imagery summarized as Normalized Difference Vegetation Indices [henceforth “NDVI”]; [ 15,16] for 12 individual months in the year spanning from April 1992 – March 1993, which was the period of availability of imagery closest to the period of plague transmission characterized by our occurrence data (a single year was used owing to limited availability of monthly data AVHRR sets). The NDVI data layers that we used are estimators of the photosynthetic mass presented within 1 km grid cells across the landscape [17]. We used individual monthly NDVI values to provide the model with information about seasonal variation in ‘greenness,’ which has been seen to be important in previous exercises in prediction of disease transmission geography [18], [19]. Although clearly other means of summarizing seasonality in greenness of landscapes are available [e.g., 20], the data sets and methodologies described in the latter paper have not been made broadly available.

Second, we explored the utility of fine-resolution interpolated climate dimensions [21]. These data, consisting essentially of monthly temperature and precipitation values averaged over 1950–2000, have been processed into 19 ‘bioclimatic variables’ that are thought to be more biologically relevant than the raw values [21]. Because of suspected high levels of intercorrelation among these variables, we explored correlations among them, and eliminated one member of each variable pair that showed high correlations, choosing the particular variable to eliminate based on ease of interpretation of the variable, leaving the following variables for analysis: annual mean temperature, mean diurnal range, maximum temperature of warmest month, minimum temperature of coldest month, annual precipitation, precipitation of wettest month, and precipitation of driest month.

**Figure 1:**
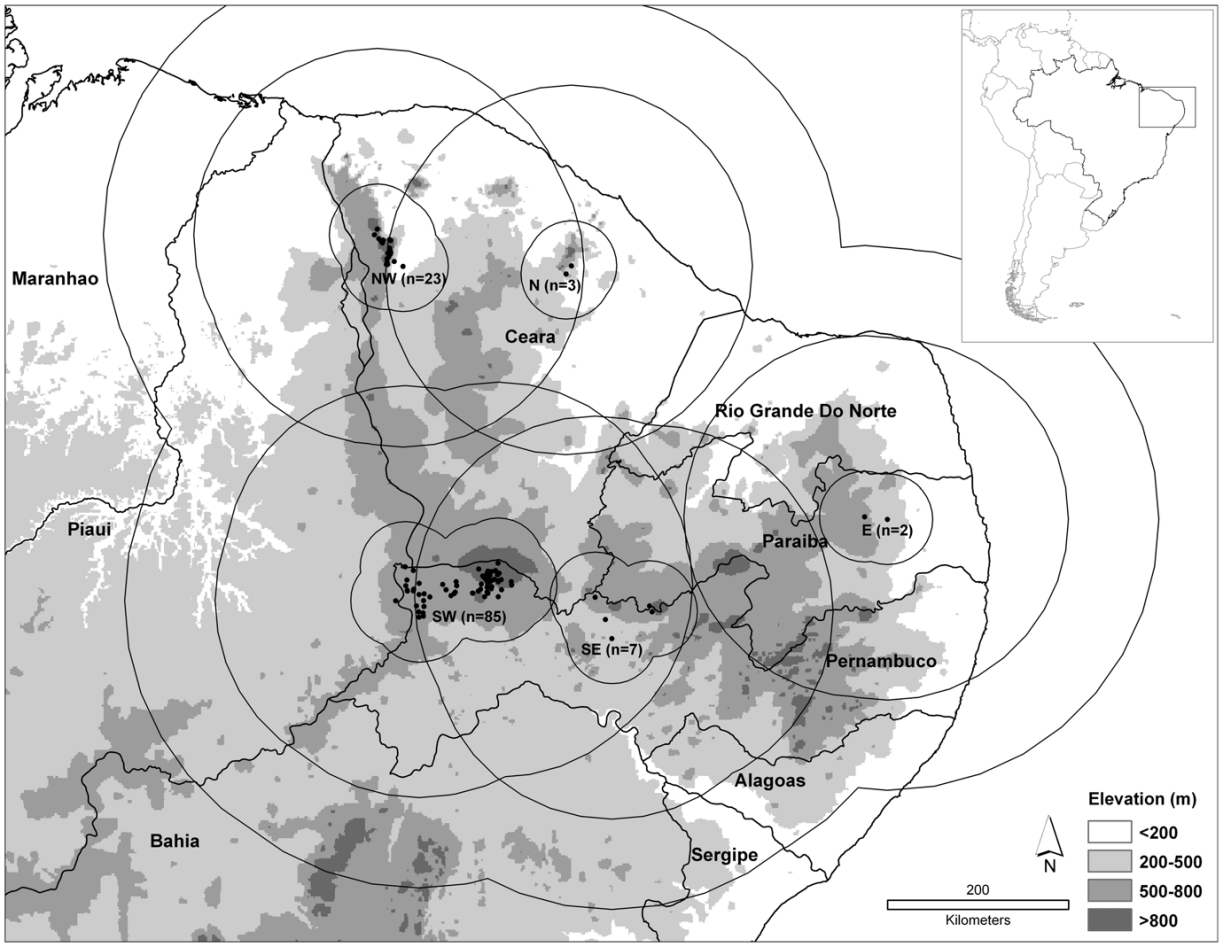
Overview of the area of analysis in northeastern Brazil. Inset shows geographic location. Map shows the five plague foci in the region and known plague occurrences (black dots), each with its respective 50 km and 200 km testing regions (see Methods). Shading indicates elevation, as follows: white  = <200 m, light gray = 200–500 m, medium gray = 500–800 m, and dark gray = >800 m.

Based on initial testing, we also explored inclusion of elevation as a predictor layer. A digital summary of elevational variation across the study area was obtained from the Hydro-1K data set [22], with 1 km native spatial resolution. In sum, we analyzed NDVI and climate data sets, each with and without elevation data. All layers were clipped to the area within 300 km of known plague occurrences for analysis (Figure 1).

### Ecological Niche Modeling

Methods and approaches for estimating ecological niches from species' occurrence data have seen considerable exploration in recent years [23], [24]. Outcomes of these tests have been mixed, with some serious criticisms of the algorithm used herein, the Genetic Algorithm for Rule-Set Prediction (GARP) [25]—these criticisms [23], [26], however, have been based either on misunderstandings of how to use the algorithm [27] or on artifactual differences in performance measures [28], [29]. In reality, and when properly used and evaluated, GARP offers estimates of species' ecological niches that are highly robust to small sample size and to broad gaps in spatial coverage of landscapes in terms of input data [28], [29]—for this reason, we used this approach throughout this study.

GARP is an evolutionary-computing method that estimates niches based on non-random associations between known occurrence points for species and sets of GIS coverages describing the ecological landscape. Occurrence data are used by GARP as follows: 50% of occurrence data points are set aside for an independent test of model quality (extrinsic testing data), 25% are used for developing models (training data), and 25% are used for tests of model quality internal to GARP (intrinsic testing data). Distributional data are converted to raster layers, and by random sampling from areas of known presence (training and intrinsic test data) and areas of ‘pseudoabsence’ (areas lacking known presences), two data sets are created, each of 1250 points; these data sets are used for rule generation and model testing, respectively.

The first rule is created by applying a method chosen randomly from a set of inferential tools (e.g., logistic regression, bioclimatic rules). The genetic algorithm consists of specially defined operators (e.g. crossover, mutation) that modify the initial rules, and thus the result are models that have “evolved”—after each modification, the quality of the rule is tested (to maximize both significance and predictive accuracy) and a size-limited set of best rules is retained. Because rules are tested based on independent data (intrinsic test data), performance values reflect the expected performance of the rule, an independent verification that gives a more reliable estimate of true rule performance. The final result is a set of rules that can be projected onto a map to produce a potential geographic distribution for the species under investigation.

Because each GARP run is an independent random-walk process, following recent best-practices recommendations [30], for each environmental data set (see above), we developed 100 replicate random-walk GARP models, and filtered out 90% based on consideration of error statistics, as follows. The ‘best subsets’ methodology consists of an initial filter removing models that omit (omission error  =  predicting absence in areas of known presence) heavily based on the extrinsic testing data, and a second filter based on an index of commission error ( = predicting presence in areas of known absence), in which models predicting very large and very small areas are removed from consideration. Specifically, in DesktopGARP, we used a “soft” omission threshold of 20%, and 50% retention based on commission considerations; the result was 10 ‘best subsets’ models (binary raster data layers) that were summed to produce a best estimate of geographic prediction. We took as a final ‘best’ prediction for each species that area predicted present by any, most, or all 10 of these best-subsets models.

Predictive models of disease occurrence may be good or bad, but model quality can be ascertained only via evaluation with independent testing data, preferably which are spatially independent of the training data to avoid problems caused by spatial autocorrelation and nonindependence of points [28]. Because only data documenting presence of plague cases were available for this study (i.e., no data were available to document that plague was *absent* at particular sites), we used a binomial probability approach to model validation: we compared observed model performance to that expected under a null hypothesis of random association between model predictions and test point distribution. Because such tests require binary (i.e., yes-no) predictions, our first step was to convert raw (continuous) predictions to binary predictions. We considered three distinct thresholds: areas predicted as suitable by any (i.e., ≥1) of the 10 replicate best-subsets models (ANY), areas predicted as suitable by most (i.e., >5) of the 10 replicate best-subsets models (MOST), and areas predicted as suitable by all of the 10 replicate best-subsets models (ALL).

In the binomial tests, the number of test points was used as the number of trials, the number of correctly predicted test points as the number of successes, and the proportion of the study area predicted present as the probability of a success if predictions and points were associated at random [31]. All testing was carried out in a series of spatially stratified tests that are detailed below. These tests evaluated the ability of models to anticipate plague case distributions across unsampled areas, considering a model as validated if it predicts case distributions better than a “model” making random predictions. As such, these tests are considerably more stringent than simple random partitions of occurrence data or cross-validation exercises.

In view of the odd, focal distribution of northeastern Brazilian plague cases (Figure 1), we carried out a series of tests of predictive abilities of models among the five foci that are easily discernable. In each case, we examined model predictivity in a *k* – 1 framework: with *k* = 5 foci, we tested all combinations of 4 foci by means of their ability to predict spatial distributions of plague cases in the fifth focus. Tests were developed within two spatial contexts—within 50 km and within 200 km—surrounding the known occurrences within the target focus.

Finally, we wished to develop a single overall model that represents the best-available picture of plague case-occurrence risk across northeastern Brazil, albeit not including statistical testing as above. This model was built using all occurrence data available. To assess uncertainty in these predictions based on all case-occurrence information, we built 100 models each based on a random 50% of the occurrence data chosen at random without replacement. These models thus capture the degree to which plague case-occurrence data availability may drive the results of the analyses, and we consider areas that are predicted consistently in all of these replicate analyses as most certain. We projected this model onto environments across eastern Brazil to provide a broader-extent visualization of the ‘niche’ of plague in northeastern Brazil.

### Niche Characterization

To explore environmental factors associated with positive and negative predictions of suitability for DF transmission, we explored further the environmental correlates of the model based on all points. We plotted 1000 points randomly across areas of the municipalities predicted as absent or present by this model. We then assigned the value of each input environmental and topographic layer to each of the random points, and exported the associated attributes table in DBF format, which was then used for comparisons of environmental characteristics of areas predicted as suitable and unsuitable.

## Results

**Figure 2:**
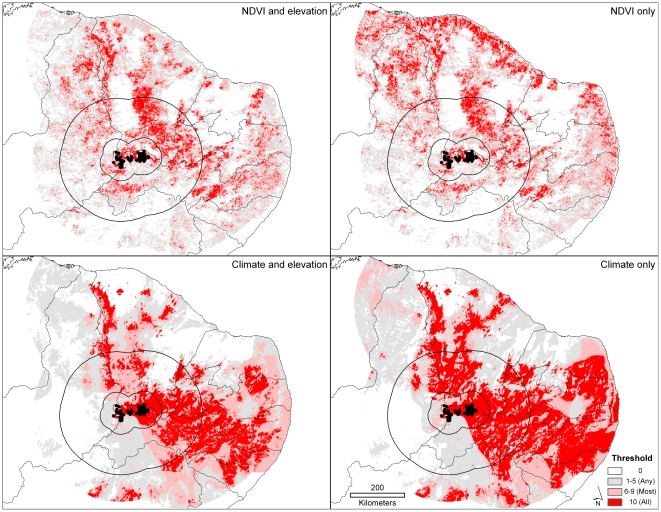
Example of model predictions for plague suitability in northeastern Brazil. The maps show the results of using occurrence data from four plague foci to predict the distribution of cases in the southwest focus, based on climate data and AVHRR NDVI greenness index data, with and without elevation data. 50 km and 200 km testing extents are shown. Shading indicates the number of best-subset models (see Methods) that predict suitability- light gray = any (1–5), pink = most (6–9), and red = all (10).

The focal and discontinuous nature of plague case distributions in northeastern Brazil is at once visible in the raw distribution of the occurrence points derived at the outset of this study (Figure 1). The discontinuities that have been assumed based on the clusters of known occurrences are supported by our ecological niche models, many of which show relatively small areas of highly suitable conditions separated by less-suitable areas (see, e.g., Figure 2). What is more, this result is manifested with or without elevation included in the analysis, and thus is not a simple consequence of topographic differences; it is also manifested in analyses based on both surface reflectance (NDVI) and climatic variables. As such, we interpret the discontinuity of plague distributions in northeastern Brazil as dependent on a multidimensional suite of environmental variables.

The model predictions in general performed quite well in anticipating plague case distributions in areas not included in model training. That is, plague not only occurs in discontinuous foci, but it also occurs under predictable and circumscribed environmental conditions, which is the basis for the success of the niche model predictions. The broadest panorama of results shows significant results dominating in the southwestern and northwestern foci (Table 1). However, the frequency of significant results in these tests is clearly and linearly related to sample size on a log_10_ scale (*P*<0.05), suggesting that predictivity would be excellent throughout the region were sample size distributions to be more adequate.

**Table 1:** Summary of results of binomial tests of model predictions at 50 km and 200 km extents.

			50 km		200 km	
Region	Threshold	Predictive success	Prop. Area	Binom. Prob.	Prop. Area	Binom. Prob.
**NDVI with Elevation**						
SW	any	84/85	0.754	**<0.001**	0.664	**<0.001**
SW	most	60/85	0.369	**<0.001**	0.337	**<0.001**
SW	all	26/85	0.136	**<0.001**	0.125	**<0.001**
SE	any	7/7	0.967	0.793	0.813	0.234
SE	most	7/7	0.839	0.292	0.654	0.051
SE	all	6/7	0.550	**0.015**	0.424	**0.002**
E	any	2/2	0.789	0.623	0.670	0.449
E	most	2/2	0.333	0.111	0.307	0.094
E	all	0/2	0.087	0.167	0.100	0.189
NE	any	3/3	0.964	0.895	0.847	0.608
NE	most	3/3	0.770	0.456	0.592	0.207
NE	all	1/3	0.420	0.381	0.341	0.270
NW	any	23/23	0.910	0.115	0.840	**0.018**
NW	most	21/23	0.548	**<0.001**	0.452	**<0.001**
NW	all	16/23	0.257	**<0.001**	0.152	**<0.001**
**NDVI without Elevation**						
SW	any	73/85	0.656	**<0.001**	0.585	**<0.001**
SW	most	53/85	0.297	**<0.001**	0.283	**<0.001**
SW	all	21/85	0.105	**<0.001**	0.100	**<0.001**
SE	any	7/7	0.948	0.689	0.782	0.179
SE	most	7/7	0.772	0.163	0.616	**0.034**
SE	all	5/7	0.467	**0.044**	0.406	**0.002**
E	any	2/2	0.699	0.488	0.663	0.439
E	most	2/2	0.407	0.165	0.419	0.176
E	all	0/2	0.193	0.348	0.243	0.426
NE	any	3/3	0.951	0.861	0.885	0.694
NE	most	3/3	0.867	0.651	0.749	0.420
NE	all	2/3	0.694	0.335	0.609	0.226
NW	any	23/23	0.974	0.549	0.944	0.263
NW	most	20/23	0.842	0.273	0.769	0.073
NW	all	18/23	0.587	**0.014**	0.550	**0.006**
**Climate with Elevation**						
SW	any	85/85	0.984	0.250	0.746	**<0.001**
SW	most	74/85	0.631	**<0.001**	0.470	**<0.001**
SW	all	62/85	0.405	**<0.001**	0.232	**<0.001**
SE	any	7/7	0.998	0.985	0.926	0.585
SE	most	7/7	0.881	0.413	0.527	**0.011**
SE	all	7/7	0.590	**0.025**	0.206	**<0.001**
E	any	1/2	0.557	0.311	0.560	0.313
E	most	1/2	0.371	0.138	0.309	0.095
E	all	0/2	0.169	0.309	0.163	0.300
NE	any	3/3	0.773	0.462	0.461	0.098
NE	most	3/3	0.138	**0.003**	0.200	**0.008**
NE	all	0/3	0.033	0.097	0.085	0.234
NW	any	20/23	0.425	**<0.001**	0.259	**<0.001**
NW	most	15/23	0.356	**0.001**	0.169	**<0.001**
NW	all	2/23	0.266	0.965	0.126	0.569
**Climate without Elevation**						
SW	any	84/85	0.989	0.398	0.783	**<0.001**
SW	most	74/85	0.670	**<0.001**	0.492	**<0.001**
SW	all	71/85	0.477	**<0.001**	0.357	**<0.001**
SE	any	7/7	1.000	1.000	0.918	0.550
SE	most	7/7	0.982	0.878	0.712	0.092
SE	all	7/7	0.687	0.072	0.241	**<0.001**
E	any	1/2	0.497	0.247	0.506	0.256
E	most	0/2	0.365	0.597	0.322	0.541
E	all	0/2	0.000	**<0.001**	0.000	**<0.001**
NE	any	3/3	0.791	0.495	0.467	0.102
NE	most	3/3	0.240	**0.014**	0.259	**0.017**
NE	all	1/3	0.003	**<0.001**	0.022	**0.001**
NW	any	19/23	0.410	**<0.001**	0.217	**<0.001**
NW	most	16/23	0.341	**<0.001**	0.147	**<0.001**
NW	all	14/23	0.269	**<0.001**	0.070	**<0.001**

**Figure 3:**
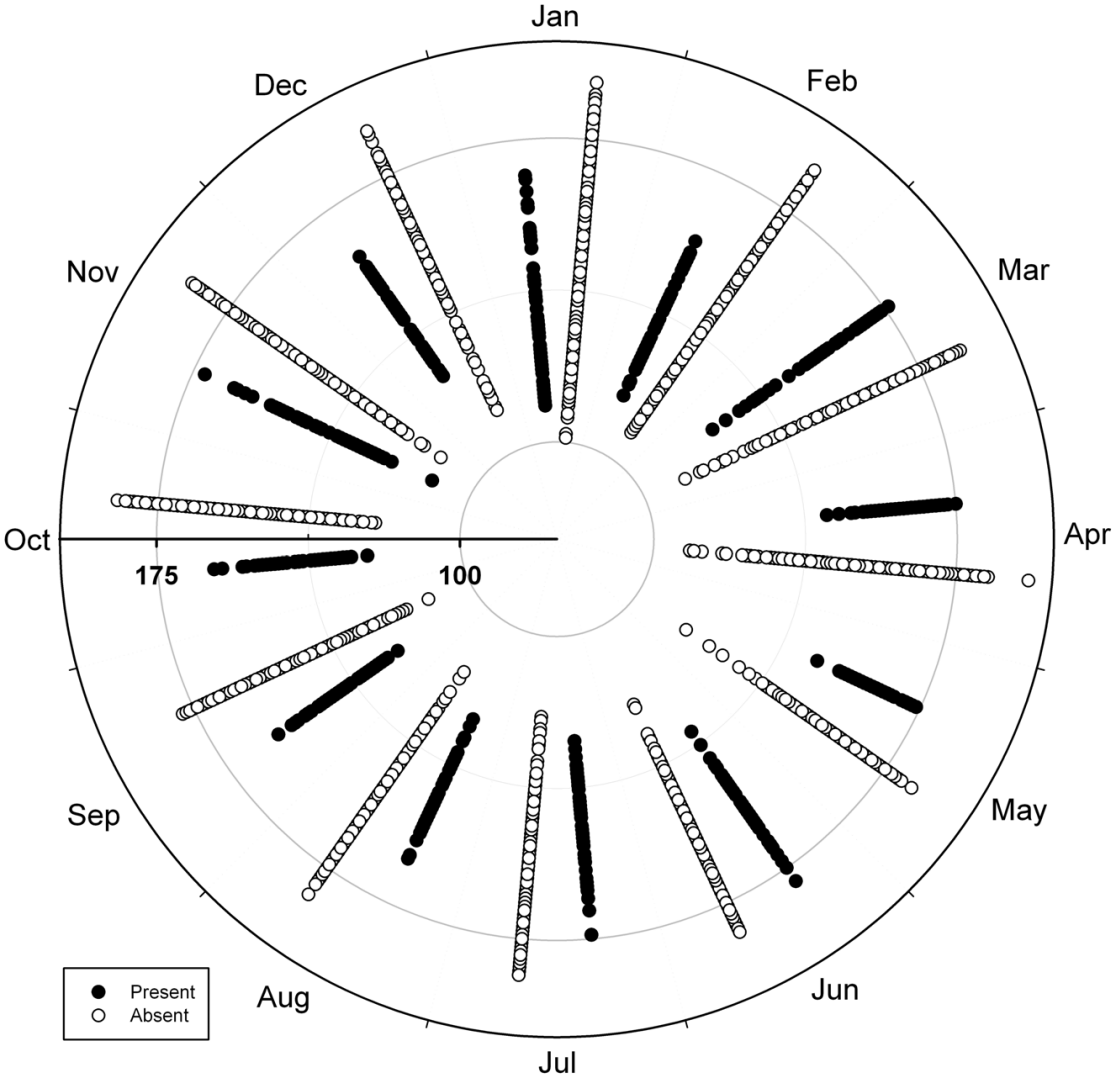
Visualization of year-round trends in AVHRR NDVI greenness index for plague-suitable and unsuitable areas. Areas predicted as suitable (i.e., suitability value of 10) versus unsuitable (i.e., suitability value of 0) for plague transmission are contrasted.

Finally, visualizing plague distributions in environmental dimensions (Figure 3), we see clear differences in the seasonal pattern of variation in greenness between areas predicted as suitable (i.e., suitability value of 10) and those predicted as unsuitable (value 0). That is, no marked seasonal variation is notable in areas predicted as unsuitable, whereas areas predicted as suitable show a marked elevation in greenness in April and May, and lower values thereafter, probably corresponding to patterns of rainfall (i.e., rainy season beginning in March, and ending by August).

**Figure 4:**
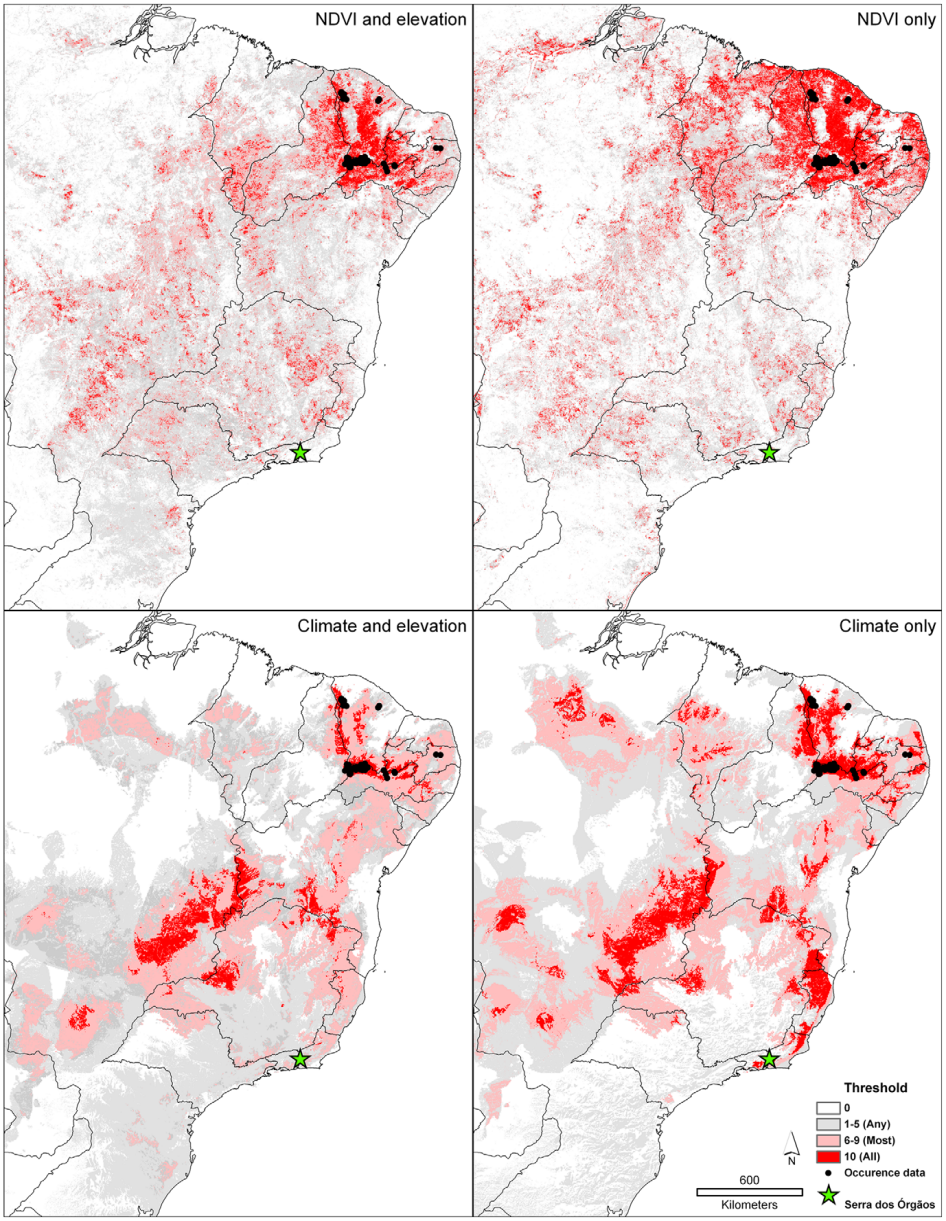
Projection of models based on all available occurrence data from northeastern Brazil across eastern Brazil. The green star represents the approximate location of the Serra dos Orgãos plague focus. Shading indicates the number of best-subset models (see Methods) that predict suitability- light gray = any (1–5), pink = most (6–9), and red = all (10).

Extending the model predictions across broader areas—namely all of northeastern and eastern Brazil—yields a picture of potential plague distribution across the region (Figure 4). Because plague transmission to humans in Brazil is currently nil, and no broad-extent data are available regarding circulation among mammals, we have few means of testing the reality of these model projections. However, at least in the case of models based on climatic dimensions, the area predicted as suitable includes the Serra dos Orgãos sites from which plague has been documented [5], [32].

## Discussion

The models that we developed for Brazilian plague offer several intriguing insights into plague distribution, ecology, and natural history in Brazil. However, understanding the limitations of these models is critical, prior to any detailed interpretation or exploration. First and foremost among the limitations of this study are the occurrence data used as input: we relied on human case-occurrence reports accumulated by the Serviço Nacional de Referência em Peste do Centro de Pesquisas Aggeu Magalhães and published in diverse scientific publications [6], [7], [8], [9], [13]. Our use of these data thereby assumes that human case-occurrences are representative of the ecological and environmental situations under which plague is maintained in the zoonotic world, which may not be the case, given the long chain of events necessary for a zoonotic occurrence to be represented in our data set (i.e., transmission to human, correct diagnosis, international reporting). On a finer scale, we also make the not-completely-satisfactory assumption that that place of residence (at the level of the ranch or settlement) is representative of the site of infection, which may be variably true depending on the particular social network and local economy.

One point that became clear in our analyses, confirming previous opinions, is that plague has a highly discontinuous and focal distribution in northeastern Brazil. Our initial suspicions that elevation played a significant role in creating these ‘islands’ were not supported, as analyses with and without elevation included as a predictor variable reconstructed the insular nature of the distribution. The NDVI-based analyses are particularly instructive, as they have no direct, mathematical relation to elevation [as do climate interpolations; 21]—rather, the discontinuous plague distribution in northeastern Brazil appears to reflect multidimensional qualities of the landscape and environment (which of course may be related biologically to elevation), rather than any simple univariate causation.

Previous studies had attributed the cause of plague focality in Brazil to elevation [1]. Baltazard [1] emphasized that Brazilian plague foci are independent—that is, that transmission appears to occur in uncorrelated patterns in different foci. Baltazard [1] also pointed out that these foci are all in elevated areas, and that they are subject to distinct precipitation regimes. Although plague has frequently shown long periods of apparent inactivity (i.e., no human cases), its reappearance at intervals nonetheless indicates its long-term persistence.

The foci are limited geographically, although their footprint can appear to expand during major outbreaks. These expansions appear to correspond to periods of particularly favorable conditions for plague transmission in the highland area, spreading out via valleys into the surrounding lowland areas. If these favorable conditions persist, taking the form of a prolonged winter, rodent host reproduction may be elevated, and plague may be able to spread beyond the limit of the highland areas into the dry *sertão* per se. This line of thinking led Baltazard [1] to consider the plague foci of Serra da Ibiapaba, Serra do Baturité, Serra do Machado, Serra de Uruburetama, Serra da Pedra Branca, Serra das Matas in northern Ceará (see Figure 1) as a single focus. Vieira and Coelho [4], in contrast, argued that these foci should be treated as isolated and independent. Our analyses suggest that these foci are dependent on a broad suite of conditions, and are not simple or direct correlates of elevation.

Another factor that may play in the picture of focality is the presence of key rodent hosts for plague, including *Necromys lasiurus* (formerly placed in *Bolomys* and *Zygodontomys*). *Necromys* is the rodent that is most abundant in northeastern Brazilian plague foci, and was considered as responsible for causing epizootics, from which the infection spreads to other species [1]. Given the distribution of this species, other species of rodents must be involved in plague maintenance farther south, for example in the Serra dos Órgãos, Rio de Janeiro state, Brazil. The relative roles of the distribution of the rodent hosts and the fleas (*Polygenis* spp.) remain to be evaluated in detail.
